# Combined fluticasone furoate/vilanterol reduces decline in lung function following inhaled allergen 23 h after dosing in adult asthma: a randomised, controlled trial

**DOI:** 10.1186/2045-7022-2-11

**Published:** 2012-06-27

**Authors:** Amanda Oliver, Dean Quinn, Caroline Goldfrad, Benjamin van Hecke, Jonathan Ayer, Malcolm Boyce

**Affiliations:** 1 GlaxoSmithKline Respiratory and Immuno-Inflammation Medicines Development Centre, Stockley Park, London, UK; 2 P3 Research, Wellington, New Zealand; 3 Hammersmith Medicines Research Ltd, London, UK; 4 Current address: GSK Medicines Research Unit, Prince of Wales Hospital, Randwick, Australia

**Keywords:** Asthma, Early allergic response, ICS, LABA, Once-daily

## Abstract

**Background:**

There is a need for preventative asthma maintenance therapy that provides lasting bronchoprotection against allergen provocation. Fluticasone furoate (FF) is a novel inhaled once-daily corticosteroid, being investigated as monotherapy for asthma and in combination with vilanterol (VI), a novel inhaled once-daily long-acting beta-agonist, for asthma and chronic obstructive pulmonary disease.

**Methods:**

In a crossover study of 52 subjects with mild asthma, FF/VI 100/25mcg and FF 100 dosed once-daily in the evening for 28 days were compared with placebo to evaluate their capacity to provide bronchoprotection against the early asthmatic response (EAR) stimulated by an inhaled allergen challenge. Bronchoprotection was assessed by change from post-saline baseline in weighted mean (wm) forced expiratory volume in 1 s (FEV_1_) for the first 2 h post-allergen challenge, which was on Day 29 (22–23 h post final dose on Day 28). The EAR was also assessed using maximum percent decrease from post-saline baseline and minimum absolute FEV_1_; the incidence of adverse events was a secondary endpoint.

**Results:**

FF/VI 100/25 and FF 100 both provided significant bronchoprotection against the EAR for all endpoints assessed. For wmFEV_1_ over the first 2 h post-allergen challenge, a 162 mL (95% CI, 87 to 237 mL) difference was observed between placebo and FF 100, while a 145 mL (95% CI, 69 to 222 mL) difference was observed between placebo and FF/VI 100/25 treatment. No difference between active treatments was observed (−17 mL; 95% CI, –91 to 57 mL). Both treatments were well tolerated.

**Conclusions:**

FF 100 alone and in combination with VI 25 provides significant bronchoprotection against the EAR in subjects with mild asthma. That this protection is provided at the trough of dosing, i.e. 23 h post last dose, supports the utility of FF 100 and FF/VI 100/25 as viable once-daily therapies.

**Trial registration:**

Clinicaltrials.gov identifier: NCT01128569, GSK Study number: HZA113090

## Background

The link between asthma and atopy is well established [1]. Many asthmatic subjects exhibit an asthmatic response (AR) to aeroallergens [2-4] that can lead to a reduction in patients’ quality of life and be an important trigger of exacerbations [5]. Experimental allergen challenge studies have shown the AR to comprise two temporal events, of which some subjects experience both while others experience only one [6]. The early AR (EAR) starts about 20 min after exposure to allergen in a sensitized subject and is typified by a rapid decline in lung function, which recovers within 2–3 h. It has been shown to be most associated with immunoglobulin E-mediated mast cell activation and release of spasmogenic mediators [6-8]. The late AR (LAR) consists of a subsequent, less acute, decline in lung function starting between 2–4 h post-exposure, which is most severe at 8–12 h, and which may not be fully recovered by 24 h. Experimentally, the LAR has been shown to be characterised by an inflammatory infiltrate; subjects who develop the LAR also exhibit increased airway hyper-responsiveness (AHR) for several days [3-8].

The LAR, when initiated by allergen challenge, is recognized as a valid clinical model of asthma, and has been widely employed to test the mechanism of the response [3,4], and the efficacy of interventions [9-11]. The LAR is regarded as more clinically important than the EAR [12] and is also more amenable to currently available therapy, as attenuation of the LAR is consistently demonstrated with ICS [3]. The EAR nonetheless represents an important clinical event in sensitized asthmatic subjects, as it can result in a significant decline in lung function, albeit of a relatively short duration, and can also contribute to worsened AHR if it occurs in combination with the LAR [3]. Published literature suggests that regular therapy with ICS can reduce the early asthmatic response (EAR) [13,14] though this has not been consistently observed [10] suggesting a need for improved therapies. The goal of asthma management is to achieve optimal control, which includes normal lung function and the need to use rescue therapy less than twice a week [1]. Exposure to aeroallergens may result in an EAR in sensitized individuals, which in turn may lead to symptoms and necessitate the use of rescue medication. As such it is important that the EAR, in addition to the LAR, be limited or abrogated by maintenance therapy.

Current maintenance therapy for asthma typically comprises an inhaled corticosteroid (ICS), supplemented, where additional control is required, with additional controller medications, the preferred add-on therapy being an inhaled long-acting beta-agonist (LABA). Fluticasone furoate (FF) is a novel once-daily ICS that is efficacious and safe in mild-to-moderate asthma [15-18]. Vilanterol (VI), a novel inhaled once-daily LABA also exhibits efficacy and safety in persistent asthma [19-21]. This study sought to explore the impact of the novel ICS, FF, on the EAR at the trough of once-daily dosing (i.e. 22–23 h post-dose), and to determine whether addition of VI to FF had any additional effect. This approach differs from the majority of allergen-response studies, where the efficacy of treatment is assessed in the challenge model shortly after dosing or at peak drug levels.

## Methods

### Design

This was a multi-centre, randomised, double-blind, placebo-controlled, 3- period crossover study conducted at two sites in the UK, one in Germany and one in New Zealand. Recruited subjects were screened within 14 to 42 days before the first dose of study treatment, with a run-in period of at least 14 days before the first dose of study medication. During screening, at which time the subject’s specific allergen and dose of allergen were established (see “Allergen challenge” section in Methods), and after successful completion of the run-in period, subjects were randomised by RandAll (GlaxoSmithKline validated internal randomisation software) to 1 of 6 treatment sequences, each comprising 3 treatment periods. Over the 3 periods, each subject took 28 days of once-daily inhaled study treatment (FF 100mcg, FF/VI 100/25mcg or placebo) via a novel dry-powder inhaler each evening between 6 pm and 8 pm. Subjects were dosed in the evening on the basis of evidence that FF once-daily evening dosing resulted in similar efficacy to twice-daily dosing at half the evening dose, while morning dosing although effective resulted in numerically smaller improvements [18]. On Day 29 subjects underwent an inhaled allergen challenge 22–23 h after their Day 28 treatment, thus the challenge was initiated between 4 pm and 7 pm. The morning following the challenge subjects were sent home providing their FEV_1_ was restored to >90% of post-saline baseline. A washout period of 21–35 days was required between treatment periods. Following completion of the last treatment period, there was a follow-up period of 7–21 days.

### Allergen challenge

The subject-specific allergen was chosen based on the relative size of awheal reaction to cat hair, house dust mite and grass pollen allergens. The skin prick test was administered either at screening or within 12 months before starting the study. The dose of allergen used was determined during screening using an allergen challenge where subjects were required to inhale increasing doses of allergen using a dosimeter and the effect on FEV_1_ was measured after each inhalation. Prior to the allergen challenge, a post-saline FEV_1_ was obtained following a saline inhalation (post-saline baseline). The presence of an EAR was determined by a fall in FEV_1_ of ≥20% from the post-saline baseline value between 5 and 30 min after the final concentration of allergen. The presence or absence of the LAR was not determined. On Day 29 of each treatment period, subjects underwent inhaled allergen challenges 22–23 h (between 4 pm and 7 pm) after their Day 28 treatment using a bolus dose of allergen, which was calculated by totalling all the allergen administered during the screening allergen challenge. Subjects were required to stay in the unit overnight for reasons of safety and were sent home the following day providing their FEV_1_ was restored to >90% of the post-saline baseline value. A detailed description of the screening and bolus allergen challenge procedures used in this study is provided in Additional file 1.

### Patients

To be eligible for study entry, subjects were required to be aged 18–65 years and to have a documented history of bronchial asthma for at least 6 months, managed with intermittent inhaled short-acting beta-agonist (SABA) therapy only. Subjects were required to exhibit a pre-bronchodilator FEV_1_ of >70% at screening, and had to show a methacholine challenge PC_20_ of <8 mg/mL at screening, or previous adenosine monophosphate or histamine challenge PC_20_ values of <60 mg.mL^-1^ or <8 mg.mL^-1^, respectively, within the last 6 months. Subjects had to be current non-smokers who had not used any tobacco products in the 6-month period preceding the screening visit and who had a pack history of ≤10 pack years (number of pack years = number of cigarettes per day/20 x number of years smoked). In addition, subjects were required to demonstrate a positive skin prick test (defined as wheal ≥3 mm, relative to the negative control) to one or more of a range of allergens. At screening, subjects were required to exhibit an EAR to inhaled allergen challenge (see Additional file 1). Finally, to commence each treatment period, subjects were required to exhibit an FEV_1_ value within 10% of the value exhibited at screening. Exclusion criteria from the study included the presence of a respiratory tract infection or asthma exacerbation within 4 weeks before the administration of the first study dose, and a history of life-threatening asthma, defined as an asthma episode that required intubation and/or any of hypercapnea, respiratory arrest and hypoxic seizures. Subjects symptomatic with hayfever at screening, or predicted to experience hayfever during days 21 to 29 of any treatment period, were also excluded, as were those with known hypersensitivity to corticosteroids or beta-agonists. All patients provided written informed consent, and the study was approved by local institutional review boards and regulatory authorities, as appropriate.

### Outcome measures

The primary objective was to evaluate the bronchoprotective effect of treatment with repeated inhaled doses of FF/VI 100/25 and FF 100 compared with placebo on the EAR to inhaled allergen 22–23 h post-dose in subjects with mild asthma. The secondary objective was to compare the effect of FF/VI 100/25 with that of FF 100. These objectives were assessed through the primary endpoint of weighted mean (wm) change from post-saline baseline in FEV_1_ between 0–2 h after the allergen challenge on treatment period Day 29 (22–23 h post-treatment on Day 28). The secondary efficacy endpoints were: (i) maximum percentage decrease from post-saline baseline (Day 29) between 0–2 h, and (ii) minimum FEV_1_ (i.e. maximum absolute decrease from post-saline baseline [Day 29], hereafter referred to as maximum absolute decrease from post-saline baseline) over the same time period. FEV_1_ was also determined on Day 1 of each treatment period, pre-dose and on Day 29 of each treatment period prior to initiation of the allergen challenge. Summary statistics for these evaluations are presented in results. In addition, the incidence of treatment-emergent adverse events (AEs) was assessed as a secondary endpoint. The occurrence of AEs was monitored from Day 1, and serious AEs (SAEs) from screening, through to the end of follow-up. Clinical laboratory and vital signs were assessed during screening, at pre-dose on Day 1, and before the allergen challenge on Day 29 of each treatment period. A 12-lead ECG was recorded during screening only, and a physical examination was done during screening and again during follow-up.

### Statistical analysis

This study was exploratory in nature, therefore no formal hypothesis testing was performed, nor was the study powered to detect treatment differences; instead point estimates and 95% confidence intervals (CI) for treatment differences for each endpoint were calculated and the study was designed to estimate the mean treatment difference between treatment groups with a certain degree of precision. A within-subject variability of 191 mL (estimated from a previous study) and a sample size of 36 subjects would ensure the half-width of the 95% CI for the treatment difference was no larger than 88 mL. Approximately 42 subjects were to be randomised to achieve 36 completed subjects (assuming a 10% failure rate). The intent-to-treat (ITT) population comprised all randomised subjects who received at least one dose of study medication and constituted the primary population for all efficacy and safety analyses. Only protocol deviations that were considered to affect efficacy were excluded from the efficacy analyses. The primary and secondary efficacy endpoints were analysed using a mixed-effects analysis of covariance (ANCOVA) model, with fixed effects of treatment, period, subject-level baseline, period-level baseline, country, sex and age and subject fitted as a random effect.

## Results

### Subject disposition and demographics

**Figure 1:**
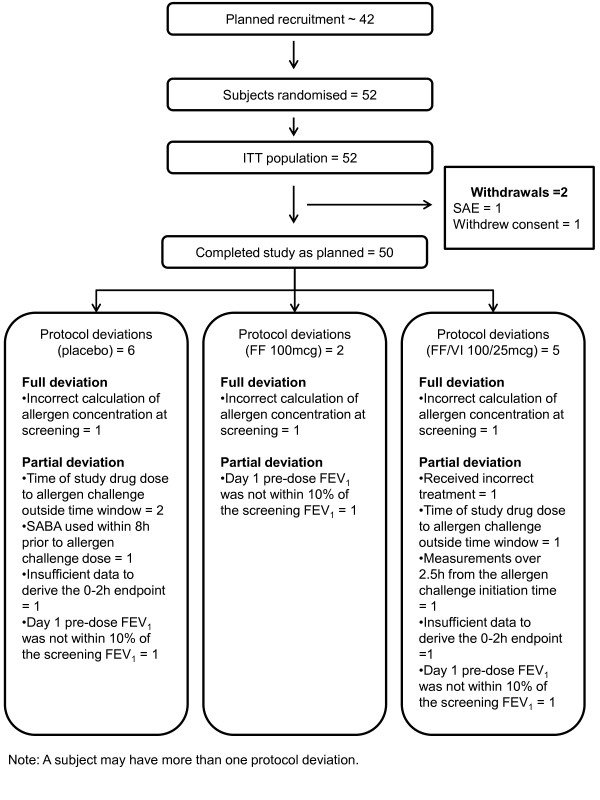
**Study CONSORT Diagram.** FF = fluticasone furoate; ITT = intent-to-treat population; VI = vilanterol

A total of 52 subjects were randomised and were included in the ITT population overall; of these subjects, 51 took part in each of the treatment periods. The number of subjects with one or more protocol deviations is illustrated in Figure 1: 45 subjects on placebo, 49 patients on FF and 46 on FF/VI did not have protocol deviations that were deemed to affect efficacy so were included in the efficacy analyses. Two subjects were withdrawn from the study, one due to a SAE during the FF 100 treatment washout; and one subject withdrew consent during the FF/VI 100/25 treatment period.

The mean subject age was 35.4 years, 35% were female, and 92% were of white race (Table 1). Baseline lung function showed a mean FEV_1_ percent predicted of 89.71% (SD = 8.85%). Ten subjects (19%) were challenged with cat dander/hair, 23 subjects (44%) with grass pollen, and 19 subjects (37%) with house dust mite. Salbutamol was the most frequently reported concomitant medication. During the study, 69, 71, and 61% of subjects reported using salbutamol in the placebo, FF 100 and FF/VI 100/25 treatment periods, respectively, and 69% reported salbutamol use during the washout periods.

**Table 1:** Demographics and Baseline Characteristics (intent-to-treat population)

**Demographics (N = 52**^**a**^**)**	
**Age, mean (SD)**	**35.4 (8.63)**
**Female, %**	**35**
**BMI, kg/m**^**2**^**, mean (range)**	**25.94 (18.9–33.6)**
**White race, %**	**92**
**Lung function**	
**Pre-bronchodilator FEV**_**1**_**, L, mean (SD)**	**3.52 (0.713)**
**Pre-bronchodilator FEV**_**1**_**% Pred., mean (SD)**	**89.71 (8.848)**

^a^51 subjects took part in each of the treatment periods. BMI = body mass index; FEV_1_ = forced expiratory volume in 1 s; L = litres; SD = standard deviation.

### Efficacy

**Figure 2:**
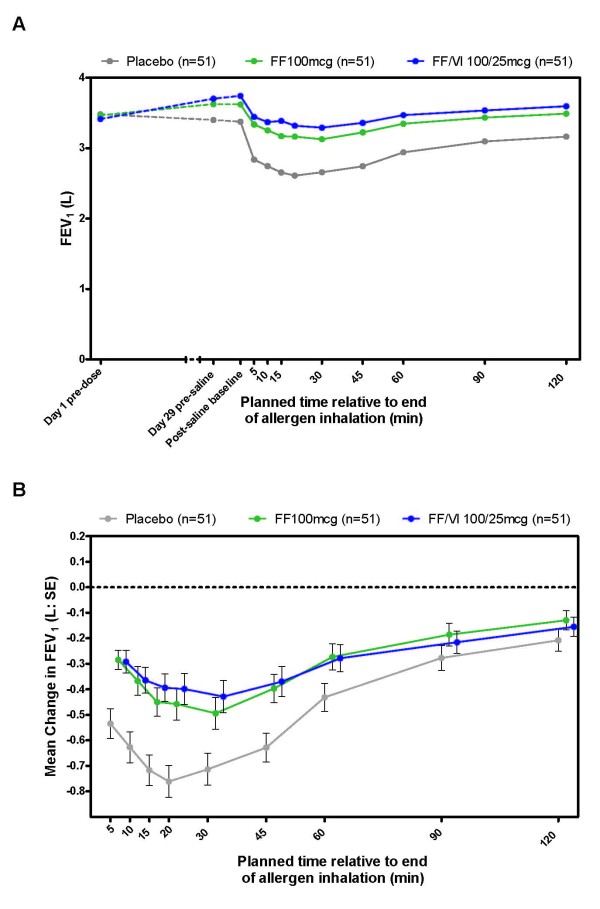
**Summary of the FEV**_**1**_**time course of mean absolute FEV**_**1**_**from Day 1 pre-dose to 2 h post allergen challenge (A) and mean FEV**_**1**_**change from allergen challenge post-saline baseline measure (B).** Therapy was dosed between 6 pm and 8 pm on Days 1–28 and the allergen challenge initiated on Day 29 (between 4 pm and 7 pm). FEV_1_ = forced expiratory volume in 1 s; FF = fluticasone furoate; L = litres; SE = standard error; VI = vilanterol. Not all subjects contributed data to all time points

A time course of absolute FEV_1_ from pre-dose on Day 1 to 0–2 h post challenge on Day 29 shows an improvement of lung function with active treatment and the characteristic decline in lung function of the EAR following allergen challenge (Figure 2a). With placebo treatment, the greatest reduction in FEV_1_ was observed between 15 and 30 min post challenge, from which point FEV_1_ increased but had not returned to the post-saline baseline level at 120 min post challenge. Relative to placebo, the immediate decline in lung function after allergen challenge was significantly reduced with FF 100 or FF/VI 100/25 treatment (Figure 2b).

Overall, significant attenuation of the EAR, as measured by all efficacy endpoints was observed with both treatments relative to placebo (Table 2). Mean treatment differences showed a 162 mL reduced decline of the wmFEV_1_ (over 0 to 2 h post-allergen challenge) for FF 100 vs. placebo and a 145 mL reduced decline of wmFEV_1_ after treatment with FF/VI 100/25 vs. placebo. No significant difference was seen between the active treatments (Table 2). The adjusted means for the wmFEV_1_ change from post-saline baseline were –372 mL (SE = 55.7), –210 mL (SE = 54.9) and –227 mL (SE = 55.0) with placebo, FF 100 and FF/VI 100/25, respectively (Additional file 2: Figure S1a).

**Table 2:** **Statistical analysis**^**a**^**of treatment differences in change from post-saline baseline (intent-to-treat population)**

	**Treatment difference**	**95% CI**
**wmFEV**_**1**_**(L)**		
**FF – placebo**	**0.162**	**0.087, 0.237**
**FF/VI – placebo**	**0.145**	**0.069, 0.222**
**FF/VI – FF**	**−0.017**	**−0.091, 0.057**
**Max. percent decrease (%)**		
**FF – placebo**	**10.951**	**8.053, 13.848**
**FF/VI – placebo**	**11.785**	**8.849, 14.721**
**FF/VI – FF**	**0.834**	**−2.010, 3.678**
**Max. absolute decrease in FEV**_**1**_**(L)**		
**FF – placebo**	**0.330**	**0.232, 0.429**
**FF/VI – placebo**	**0.331**	**0.231, 0.431**
**FF/VI – FF**	**0.001**	**−0.096, 0.097**

^a^Population sizes analysed: placebo, n = 45; FF, n = 49; FF/VI, n = 46. Efficacy endpoints were analysed using a mixed-effects analysis of covariance (ANCOVA) model, with fixed effects of treatment, period, subject-level baseline, period-level baseline, country, sex and age and subject fitted as a random effect.

CI = confidence interval; FEV_1_ = forced expiratory volume in 1 s; FF = fluticasone furoate; L = litres; VI = vilanterol; wm = weighted mean.

**Figure 3:**
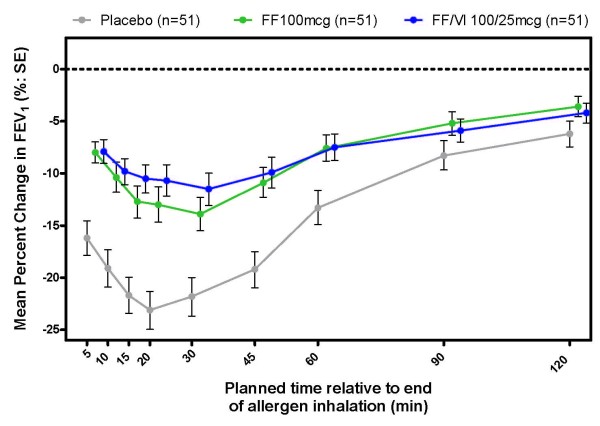
**Summary of the time course of maximum percentage change in post-saline baseline FEV**_**1**_**.** Therapy was dosed between 6 pm and 8 pm on Days 1–28 and the allergen challenge initiated on Day 29 (between 4 pm and 7 pm). FEV_1_ = forced expiratory volume in 1 s; FF = fluticasone furoate; SE = standard error; VI = vilanterol. Not all subjects contributed data to all time points

Maximum percentage change from post-saline baseline in FEV_1_ over time is shown in Figure 3, and the treatment differences between placebo, FF 100 and FF/VI 100/25 are summarised in Table 2. Significant reduction in the maximum percentage decrease from post-saline baseline was observed with each active treatment relative to placebo (11%), and no significant difference was observed between the FF 100 and FF/VI 100/25 treatments. The adjusted means for the maximum percent change from post-saline baseline in the placebo, FF 100 and FF/VI 100/25 arms were −24.991% (SE = 2.0736),–14.040% (SE = 2.0435) and −13.206% (SE = 2.0491) (Additional file 2: Figure S1b).

Differences in the maximum absolute FEV_1_ decrease from post-saline baseline between each active treatment and placebo were significant, but not significant between active treatments (Table 2). The adjusted means for the maximum FEV_1_ absolute decrease from post-saline baseline in the placebo, FF 100 and FF/VI 100/25 arms were –809 mL (SE = 77.5), –479 mL (SE = 76.5) and –478 mL (SE = 76.7) (Additional file 2: Figure S1c).

Although it was not a study endpoint, FEV_1_ was assessed pre-dose on Day 1 and prior to the allergen challenge on Day 29. Over the 28 days of dosing, a mean decline of 82.4 mL (SD = 237 mL) was observed with placebo, while increases in FEV_1_ of 146.4 mL (SD = 239 mL) and 279.4 mL (SD = 264 mL) were observed with FF 100 and FF/VI 100/25, respectively.

### Safety

On-treatment AEs were similar between the study arms, with any AE being reported in 39% of subjects during the placebo treatment period, in 43% of subjects during the FF 100 treatment period, and in 35% of subjects during the FF/VI 100/25 treatment period. Twenty-seven percent of on-treatment AEs in the placebo and FF 100 arms were deemed to be related to study treatment, and 29% of events were deemed to be related to study treatment for the FF/VI 100/25 treatment. Headache was the most frequently reported AE (Table 3). AEs occurring at a frequency of greater than 3%, and only in the active treatment arms, were: mouth ulceration and dysphonia, both of which occurred in 4% of subjects with each active treatment. Vital signs, clinical chemistry and haematology were similar between all three treatments, and no abnormalities of clinical importance were observed.

**Table 3:** Most frequent on-treatment adverse events (≥3% any treatment group) intent-to-treat population

**n (%)**	**FF 100mcg (n = 51)**	**FF/VI 100/25mcg (n = 51)**	**Placebo (n = 51)**
**Headache**	**11 (22)**	**5 (10)**	**9 (18)**
**Oropharyngeal pain**	**2 (4)**	**2 (4)**	**2 (4)**
**Nasopharyngitis**	**2 (4)**	**0**	**3 (6)**
**Cough**	**3 (6)**	**0**	**1 (2)**
**Chest discomfort**	**0**	**0**	**3 (6)**
**Mouth ulceration**	**2 (4)**	**2 (4)**	**0**
**Nausea**	**2 (4)**	**0**	**2 (4)**
**Dysphonia**	**2 (4)**	**2 (4)**	**0**
**Hot flush**	**0**	**2 (4)**	**0**
**Seasonal allergy**^**a**^	**2 (4)**	**0**	**0**

^a^One subject (2%) had seasonal allergy, reported as allergic rhinitis, during each treatment period.

FF = fluticasone furoate; VI = vilanterol.

One subject withdrew during the study due to an SAE, which occurred 4 days after the last dose in the FF 100 treatment period. This subject was provisionally diagnosed with moderate (grade 2) Still’s disease. Six weeks later, the subject was hospitalised. A diagnosis of histiocytic necrotising lymphadenitis (Kikuchi’s Disease) was made based on histology of an excised lymph node. Tapered prednisolone treatment, initiated at 60 mg per day, has been successful.

## Discussion

Both FF/VI 100/25 and FF 100 clinically and significantly suppress the EAR to an allergen challenge relative to placebo. This is supported by significant differences between active treatment and placebo being observed in (i) the wm change in FEV_1_ from post-saline baseline, (ii) the maximum percentage decrease in FEV_1_ from post-saline baseline, and (iii) the maximum absolute decrease in FEV_1_ from post-saline baseline. No major safety signals were observed in this crossover study, and in the one subject who discontinued due to an SAE, the event subsequently resolved.

Bronchoprotection against the early and late allergic response is a part of asthma control [1], and is one of the characteristics that should be expected of any asthma maintenance therapy – especially as increased levels of aeroallergens such as grasses, house dust mite or dander are associated with increased rates of asthma exacerbation and hospitalisation [5]. When the allergic response (typically the LAR) is used as a model to test the extent of bronchoprotection provided by an intervention, the test is typically conducted shortly after dosing of the intervention [10]. This approach is rational when seeking to establish whether a novel intervention is capable of providing bronchoprotection, given the strong negative predictive power of the allergen challenge model [3]. However, when considering an intervention of known efficacy, the key time to test the bronchoprotective capacity of an intervention is at the trough of dosing, as this provides evidence of the sustained effect of the intervention, as has been investigated for the twice-daily dosed ICSs fluticasone propionate and budesonide [22,23]. In the present study, the allergen challenge was initiated on Day 29, 22–23 h after the final dose of study medication, and as such total ablation of the EAR was not expected. Nevertheless, the results showed a clear and significant effect on the EAR after 28 days once-daily therapy with either FF 100 or FF/VI 100/25 yielding an approximate 150 mL reduction in the extent of the EAR over the first 2 h post-challenge relative to placebo. That this effect was also seen for maximum percentage decrease (~11%) and maximum decrease in FEV_1_ from post-saline baseline (~330 mL) suggests that even at the trough of once-daily dosing, FF 100 and FF/VI 100/25 continue to exhibit protection from allergen-induced EAR.

The secondary objective of this study was to assess the bronchoprotective effect of FF/VI 100/25 relative to FF 100. Previously it has been shown that a LABA/ICS combination resulted in significantly greater attenuation of the EAR compared to ICS alone [10], although in that study, the challenge was not conducted at the end of the dosing interval. The additional attenuation of the response provided by the LABA over the ICS response has been ascribed to ‘functional antagonism’ of the EAR/LAR by the bronchodilatory effect of the LABA [10]. LABAs have also demonstrated protection in exercise-induced asthma models [24,25] where the challenge was initiated at or near the end of the dosing period, suggesting that functional antagonism can persist throughout the dosing period. In the present study, no additional effect on suppression of the EAR was observed with FF/VI 100/25 relative to FF 100. There are a number of potential reasons for this observation. In the first instance, the mean pre-bronchodilator FEV_1_ of the subjects recruited into this study was almost 90%; therefore it is conceivable that any functional antagonism provided by VI would be obscured by the near normal baseline lung function, which was further improved after 28 days of therapy with both FF 100 and FF/VI 100/25. In the second instance, as the allergen challenge was conducted on Day 29 at trough drug levels, it is possible that any additional improvement in FEV_1_ provided by VI through functional antagonism had abated at that time point. It would be of interest to explore, in a further study, the EAR at 6, 12 or 18 h following dosing to investigate whether VI does provide functional antagonism and also the duration of this effect. However, studies assessing bronchodilation by VI over time have shown a 24h duration of effect of VI on lung function demonstrated by improvements relative to placebo in FEV_1_ measured at 24 h post-dose of 121 mL after 28 days [19] and 125 mL after 7 days [21] of dosing in subjects with persistent asthma receiving concomitant ICS.

As with all allergen challenge studies, there were strengths and limitations associated with this study. The study was limited in that only effects on the EAR were investigated, and as patients were only assessed for the presence of the EAR at screening it is not possible to know how many of those recruited were single versus dual responders. Also, while the effect of FF 100 and FF/VI 100/25 was assessed relative to placebo, the effect of VI 25mcg was not. However, a separate study (NCT01128595) investigating the effects of FF and VI alone and in combination on the EAR and LAR at 1 h post-dose in confirmed dual responders has been completed and will be reported elsewhere. A further limitation is that subjects were receiving SABA only at enrolment so the next treatment step, according to the GINA guidelines, is low-dose ICS rather than an ICS-LABA combination [1]. However, the response to inhaled allergen can be thought of as simulating the need to increase therapy, by experimentally destabilising asthma, and, therefore, mimicking a patient in need of ICS/LABA therapy. The data from this study are strengthened by a number of factors: firstly, the challenge was conducted on Day 29 of each treatment period (i.e. at the end of the dosing interval, 22-23 h post-dose on Day 28). Despite the importance of asthma control throughout the dosing interval, testing at the time of minimal drug effect has been infrequently studied in challenge models [22,23]; secondly, the design of the study, particularly the option to extend the washout period and the careful selection of subjects meant that the study was able to be conducted through the hay fever season (in the UK and Germany). Consequent to this, one subject reported two occurrences of seasonal allergy as an AE during the FF 100 treatment period, on days 2 and 23. One further subject reported allergic rhinitis on days 23, 21 and 4 of treatment periods 1, 2 and 3, respectively. It is possible that the AEs on days 21 and 23 may have influenced the primary endpoint; however, given the overall incidence of these events any effect would have been small.

## Conclusions

In conclusion, FF 100 and FF/VI 100/25 – doses that have been progressed to phase III trials – inhaled daily over 28 days provide significant and clinically relevant bronchoprotection against the EAR relative to placebo. That this protection occurs at the end of the dosing interval suggests true 24 h activity of both FF 100, and the combination of FF/VI 100/25. Consequently both have the potential to reduce the symptoms associated with the allergic response in atopic asthma subjects, and to improve asthma control and quality of life.

## Competing interests

Amanda Oliver, Caroline Goldfrad and Jonathan Ayer are shareholders and employees of GlaxoSmithKline. Dean Quinn, Benjamin van Hecke and Malcolm Boyce have no conflict of interest. Benjamin van Hecke was employed by Hammersmith Research Ltd at the time of this study.

## Authors’ contributions

All listed authors meet the criteria for authorship set forth by the International Committee for Medical Journal Editors. AO, DQ and MB developed the design and concept of the study, all authors had full access to and interpreted the data, and wrote the manuscript. AO and JA coordinated data gathering and BvH collected data. CG led the statistical analysis. All authors vouch for the accuracy and completeness of the data and the data analysis. All authors read and approved the final version of manuscript.

## Supplementary Material

Additional file 1 **Supplement 1.** Allergen Challenge, detailed method [26]. Click here for file

Additional file 2 **Supplement 2.** Change from allergen challenge post-saline baseline (least squares means, 95% CI) for wmFEV_1_**(a)**, maximum % FEV_1_ decline **(b)** and maximum absolute FEV_1_ decline **(c)**. Click here for file
