# Supplementary material for: Combined fluticasone furoate/vilanterol reduces decline in lung function following inhaled allergen 23 h after dosing in adult asthma: a randomised, controlled trial
Source: Clin Transl Allergy. 2012 Jun 27;2:11. doi: 10.1186/2045-7022-2-11 (PMC3483689; doi:10.1186/2045-7022-2-11)
Supplement: Additional file 1 — Supplement 1. Allergen Challenge, detailed method [26]. [file 2045-7022-2-11-S1.docx]

**Combined Fluticasone Furoate / Vilanterol Reduces Decline in Lung Function Following Inhaled Allergen 23h After Dosing in Adult Asthma: A Randomised, Controlled Trial**

Amanda Oliver^1^*, Dean Quinn^2^, Caroline Goldfrad^1^, Benjamin van Hecke^3^, Jonathan Ayer^1^, Malcolm Boyce^3^

1. GlaxoSmithKline Respiratory and Immuno-Inflammation Medicines Development Centre, Stockley Park, London, UK
2. P3 Research, Wellington, NZ
3. Hammersmith Medicines Research Ltd, London, UK

*Corresponding Author; Dr Amanda J. Oliver, Director, Clinical Pharmacology, Respiratory and Immuno-Inflammation Medicines Development Centre, GlaxoSmithKline, Stockley Park, UK. Tel +44 (0)20 8990 2398

Email: amanda.j.oliver@gsk.com

**Additional File: Supplement 1.**

**Allergen Challenge, detailed method**

The allergen with which a subject was challenged was determined by the skin-prick test done during or before screening and the subject’s history. The Allergen challenge was performed at screening (to determine the dose of allergen to use and to test whether the subject could demonstrate an EAR; figure a) and also at the end of each treatment period (detail below; figure b) to test the protective effects of FF/VI and FF versus placebo on attenuating the EAR. The presence of an EAR was determined on a subject-by-subject basis, at screening as follows:

All centres used the 5-breath dosimeter technique [26] to dispense the allergen at increasing inhaled doses. The occurrence of an EAR was defined as a fall in FEV_1_ of ≥20% from the post-saline baseline value within 30 minutes of allergen inhalation, and was a study entry requirement. The allergen challenge at the end of each treatment period comprised a single bolus concentration consisting of the sum of allergen concentrations given at screening. The allergen challenge was performed on Day 29 at the end of each treatment period (22–23h after the final dose of study treatment). The post-saline baseline FEV_1_ was used to establish a baseline from which to compare change in FEV_1_ during the challenge. The challenges carried out during treatment Periods 1, 2 and 3 were performed at approximately the same time as the screening challenge. During the challenge, spirometry was performed at 5, 10, 15, 20, 30, 45, 60, 90 and 120 minute post-challenge. Spirometry was performed at screening, on Day 1 before treatment; and pre-saline on Day 29.

**Screening allergen challenge (a) and bolus allergen challenge (b)**

**(a)**

**(b)**
