# Supplementary material for: Combined fluticasone furoate/vilanterol reduces decline in lung function following inhaled allergen 23 h after dosing in adult asthma: a randomised, controlled trial
Source: Clin Transl Allergy. 2012 Jun 27;2:11. doi: 10.1186/2045-7022-2-11 (PMC3483689; doi:10.1186/2045-7022-2-11)
Supplement: Additional file 2 — Supplement 2. Change from allergen challenge post-saline baseline (least squares means, 95% CI) for wmFEV1(a), maximum % FEV1 decline (b) and maximum absolute FEV1 decline (c). [file 2045-7022-2-11-S2.docx]

**Combined Fluticasone Furoate / Vilanterol Reduces Decline in Lung Function Following Inhaled Allergen 23h After Dosing in Adult Asthma: A Randomised, Controlled Trial**

Amanda Oliver^1^*, Dean Quinn^2^, Caroline Goldfrad^1^, Benjamin van Hecke^3^, Jonathan Ayer^1^, Malcolm Boyce^3^

1. GlaxoSmithKline Respiratory and Immuno-Inflammation Medicines Development Centre, Stockley Park, UK
2. P3 Research, Wellington, NZ
3. Hammersmith Medicines Research Ltd, London, UK

*Corresponding Author; Dr Amanda J. Oliver, Director, Clinical Pharmacology, Respiratory and Immuno-Inflammation Medicines Development Centre, GlaxoSmithKline, Stockley Park, UK. Tel +44 (0)20 8990 2398

Email: amanda.j.oliver@gsk.com

**Supplement 2.**

**Change from allergen challenge post-saline baseline (least squares means, 95%CI) for wmFEV_1_ (a), maximum % FEV_1_ decline (b) and maximum absolute FEV_1_ decline (c)**


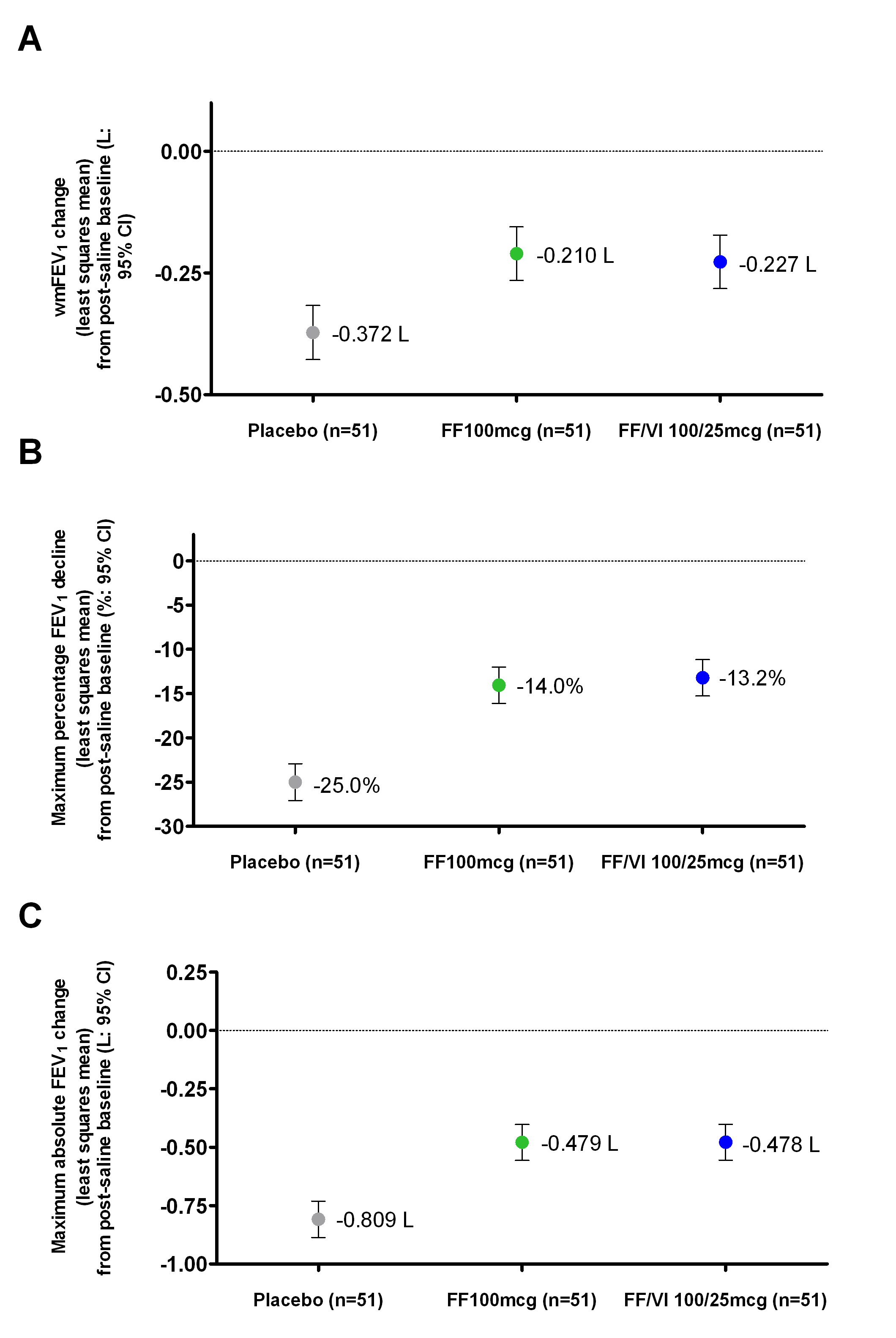


FEV_1_ = forced expiratory volume in 1 second; FF = fluticasone furoate; L = litres; VI = vilanterol; wm = weighted mean
